# Decision Making during the Learning Curve of Minimally Invasive Mitral Valve Surgery: A Focused Review for the Starting Minimally Invasive Surgeon

**DOI:** 10.3390/jcm11205993

**Published:** 2022-10-11

**Authors:** Kinsing Ko, Ad F. T. M. Verhagen, Thom L. de Kroon, Wim J. Morshuis, Leen A. F. M. van Garsse

**Affiliations:** 1 Cardiothoracic Surgery, Radboud University Medical Center, 6525 GA Nijmegen, The Netherlands; 2 Cardiothoracic Surgery, St. Antonius Hospital Nieuwegein, 3435 CM Nieuwegein, The Netherlands

**Keywords:** minimally invasive mitral valve surgery, learning curve, decision making

## Abstract

Minimally invasive mitral valve surgery is evolving rapidly since the early 1990’s and is now increasingly adopted as the standard approach for mitral valve surgery. It has a long and challenging learning curve and there are many considerations regarding technique, planning and patient selection when starting a minimally invasive program. In the current review, we provide an overview of all considerations and the decision-making process during the learning curve.

## 1. Introduction

Minimally invasive mitral valve surgery (MIMVS) has evolved rapidly since the beginning in the early 1990’s [1,2,3] and is now increasingly adopted as the standard approach for mitral valve surgery by many centers [4,5,6,7,8,9,10]. The STS database showed an increase from 15.5% (2014) to 24.6% (2018) of all mitral valve procedures in the United States being approached by a less invasive access [11]. In experienced centers, rates of minimally invasive access have been reported up to 74% [12] with good short- and long-term results in primary [4,5,6] and in redo mitral valve surgery [7,8,9,10], equal repair rates as in sternotomy approach [13,14]. In high-risk cases, such as in infective endocarditis, MIMVS showed comparable morbidity and mortality compared to sternotomy approach [15,16,17]. There are many considerations regarding the technique, planning and patient selection when starting MIMVS. In the current review, we will discuss important contributing factors for the decision-making during the learning curve of MIMVS (mini-thoracotomy or fully thoracoscopic), based on literature and our surgical experience [6,7,18].

## 2. Learning Curve of Minimally Invasive Mitral Valve Surgery

MIMVS has a long learning curve due to multiple factors complicating the learning process. The decrease of the surgical working space and remotely working with long shafted instruments while the surgical perspective is changed to a videoscopic view, is technically more demanding than working in a wide surgical field with shorter instruments. For the extra-corporeal circulation, the procedure requires peripheral canulation (femoral, axillary or direct aortic) with specific aortic clamp techniques (e.g., Chitwood clamp or endo-aortic balloon). There must be insight into the pitfalls for peripheral cannulation techniques. Adding more complex techniques, such as the endo-aortic balloon will require extra attention from the surgeon while there is already full focus needed for the surgical procedure. To train thoracoscopic skills and surgical sequence of MIMVS, training modules and simulators are available to shorten the learning curve [19,20]. The number of procedures required to complete the learning curve is reported at 75–125 cases and at least 2 surgeries per week to maintain optimal surgical outcome [12]. These numbers are based on real life data from the very beginning of MIMVS in a highly specialized high-volume center. More recently other centers have shared their experience about their learning curve and reported the same number of procedures needed to overcome the learning curve [21]. Data regarding the number of cases required to learn MIMVS with modern technology (such as a simulator) are not available yet but it is expected to be at a lower number.

## 3. Benefits and Limitations of Minimally Invasive Access

### 3.1. Benefits of Minimally Invasive Access

**Figure 1:**
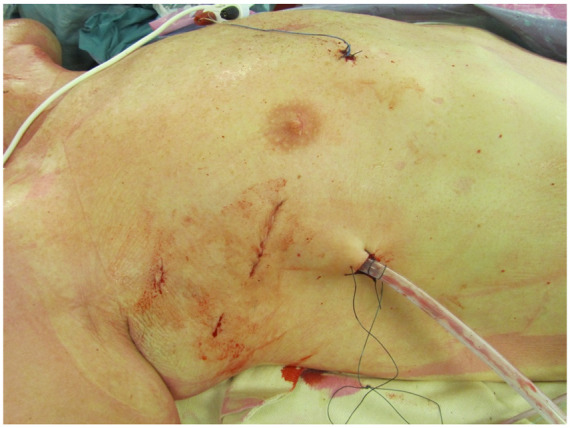
Scar of minimally invasive access for mitral valve surgery.

In literature a range of advantages is described when mitral valve surgery is performed via minimally invasive access. A reduction of blood transfusions, post- operative atrial fibrillation, ventilation time, length of stay in intensive care unit or hospital and risk of deep sternal wound complications are reported for MIMVS [22,23]. Furthermore, there is a higher patient’s satisfaction for cosmetics in MIMVS due to the location and size of the incision (Figure 1). Minimally invasive access showed improved quality of life and faster return to daily activity compared to sternotomy in the early postoperative phase (first 3 months) [24,25]. The results of the Mini-Mitral Trial in the United Kingdom will soon report their findings on differences in functional outcome (for example quality of life) in the first year after surgery [26]. Although not yet confirmed by studies, faster recovery and return to daily activities may have a positive economic impact as well. The lateral access in the chest is more directly aligned with the mitral valve, providing the surgeon excellent exposure of the mitral valve. This access is even more beneficial in patients after previous cardiac surgery via sternotomy by avoiding the risks associated with repeat sternotomy [27] and by excellent exposure of the mitral valve even in patients with prior aortic valve or root surgery.

**Figure 2:**
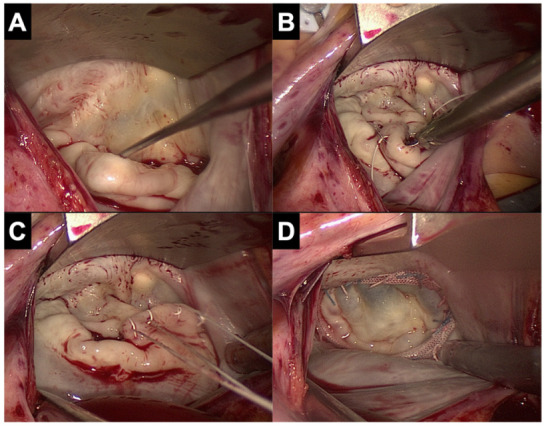
View on the mitral valve in MIMVS. (**A**) Prolapse of P2 segment. (**B**) Placement of neochords on the prolapsing segment. (**C**) Adjusting of the height of the neochords. (**D**) Result of mitral valve repair.

Unlike sternotomy, the mitral valve in MIMVS is visualized using a camera, which gives the surgical team an excellent view of the mitral valve and makes it easier for them to anticipate the next surgical steps in the procedure (Figure 2). An additional advantage of the videoscopic image is that younger colleagues can gain more insight into mitral valve pathology and repair techniques, while in sternotomy procedures they can only glimpse the mitral valve when looking over the surgeon’s shoulder. 

### 3.2. Limitations of Minimally Invasive Access

MIMVS is by no means a risk-free procedure, it has many advantages for the patients, but it carries specific risks: a potentially higher stroke risk, groin complications, aortic dissection, longer cardiopulmonary bypass and aortic occlusion time are described [22,23]. It is a high demanding technique with a challenging learning curve [12]. With the evolution of the technique and the increased experience in dedicated institutions, comparable cardiopulmonary bypass and aortic occlusion time can be achieved and a reduction of aortic dissections and strokes are reported [4,6,11,13,28]. Concomitant procedures in MIMVS are usually limited to tricuspid valve surgery, closure of defects in the atrial septum and rhythm surgery. Although concomitant aortic valve surgery [29,30] or coronary artery bypass surgery [31] are feasible through minimally invasive access, sternotomy remains the most common access for these combined procedures. Chest deformations (e.g., pectus excavatum) or adhesions in the right thorax (e.g., prior pneumothorax or trauma) can limit the view in MIMVS.

## 4. Considerations in Pre-Operative Planning and Patient Selection during the Learning Curve

### 4.1. Pre-Operative Planning and Imaging

When starting a MIMVS program, careful patient selection and planning is necessary to establish a safe and successful program. A dedicated multidisciplinary mitral valve heart team can help select the best treatment (MIMVS vs. sternotomy vs. transcatheter treatment vs. conservative) for each individual patient and is proven to improve long-term survival [32]. In addition to the standard preoperative examinations (e.g., Chest X-ray, blood tests, transthoracic and/or transesophageal echocardiography and coronary angiogram), a thoracic CT scan and aortic CT from the femoral vessels to the aortic valve provide important information for surgical planning. Based on these images, calcifications in mitral annulus, thoracic deformations, the position of the mitral valve in the thoracic cavity and distance to the sternum is objectified and the optimal position of thoracic incision can be predicted. Imaging the arterial access reveals atherosclerotic load on the ilio-femoral or axillary arteries, a possible aortic aneurysm or intra-aortic thrombus, which can help adjust surgery planning and reduce the risk of vascular and cerebral embolic complications. Attention to the venous access on CT scan will occasionally lead us to discover anatomical variants (e.g., Interrupted inferior vena cava or persistent left vena cava superior).

### 4.2. Patient Selection

Contraindications for MIMVS are safety related factors that expose unnecessary risks which are not in proportion with the benefits of less invasive access. Relative contraindications reported by the International Society for Minimally Invasive Cardiothoracic Surgery (ISMICS) are shown in Table 1 [33].

All patients with isolated mitral valve pathology should be considered for MIMVS and those with the above mentioned relative contra-indications, the advantage of MIMVS should be weighed against the operative risk. Some patients with relative contra-indications may be considered as suitable candidates for MIMVS when there is sufficient surgical experience, such as patients with morbid obesity. In these patients, the surgical exposure can be troublesome due to the increased distance from the chest to the mitral valve and elevated hemidiaphragm caused by the volume of the abdominal content. In experienced hands, these challenges can be overcome (e.g., different positioning of exposure sutures). Operating these patients in a minimally invasive fashion will even improve patient outcome since they will benefit from a faster recovery due to less surgical trauma. The ideal surgery to start with is in a non-obese patient without chest deformities, with a good left ventricular function and isolated mitral insufficiency, without aortic valve insufficiency or pulmonary hypertension and scheduled for uncomplicated repair or valve replacement. Preferably, the patient has a dominant right coronary anatomy, which would result in less ischemic injury if the circumflex artery was accidentally damaged as a result of the mitral annular sutures. Whenever the learning curve has been overcome, higher risk patients (the elderly, patients after previous sternotomy and those with morbid obesity) will actually benefit the most from a minimally invasive access [34,35].

### 4.3. Considerations of Mitral Valve Pathology during Learning Curve of MIMVS

Patients with complex repair, such as patients with anterior leaflet or bi-leaflet prolapse, should be avoided early in the learning curve as minimally invasive access should not compromise the outcome valve repair. Experienced centers have reported excellent long-term results of complex repairs (e.g., Barlow’s disease) through MIMVS with freedom from re-operation up to 93.8% in 10 years follow up and freedom from greater than 2+ grade mitral regurgitation up to 88.4% in 10 years follow up [36,37]. In these cohorts, a wide variety of repair techniques was used (leaflet resection, sliding annuloplasty, neochords, edge-to-edge repair). Mitral valve pathology with annular calcification can be challenging for valve repair or replacement, although this would be in sternotomy setting as well. Nonetheless, patients with severely calcified mitral annulus are less suitable for minimally invasive access due to the technical challenge during decalcification of the annulus and the limited access to the heart in case of complications (left ventricle dissection). Although most studies report their outcomes of patients with degenerative disease, other studies have shown good results of MIMVS in patients with endocarditis [15,16,17] and rheumatic valve disease [38].

## 5. Available Techniques and Safety Considerations

### 5.1. Safety of the Minimally Invasive Techniques

Multiple techniques are available at each step of surgery (e.g., chest access, cardiopulmonary bypass configuration, aortic occlusion). In the early days of MIMVS, there were concerns about the safety of this technique, especially with regard to aortic injury and stroke [23]. Since the 1990s, results have improved significantly due to better pre-operative planning, adjustments to surgical technique and as more experienced centers have become familiar with the technique. A recent STS database study from 2021 and recent meta-analysis show similar results for MIMVS versus sternotomy [4,5,6,7,8,9,11,39,40]. To initiate the MIMVS program, an institute should begin with the lowest risk patients with non-complex valve pathology and the most appropriate anatomy for the access. In a more advanced stage, more advanced techniques can be applied to expand the appropriate patient population that will benefit from the minimally invasive technique. However, moving towards more complex and less invasive techniques should never compromise operative safety and the valve repairability. Available techniques for each step of the operation (access, cardiopulmonary bypass, aortic occlusion) are discussed in the section below. 

### 5.2. Less Invasive Surgical Access and Visualization of the Mitral Valve

**Figure 3:**
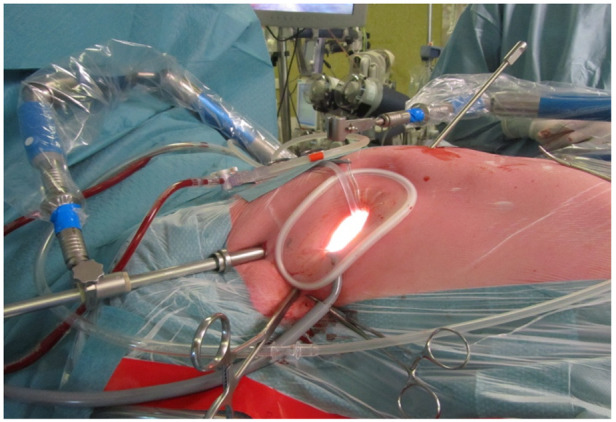
Setup of a MIMVS procedure by right anterolateral mini thoracotomy. Starting from the bottom of the soft tissue retractor in clockwise direction: Carbon dioxide insufflator, Transthoracic Chitwood clamp, video port and left atrial retractor.

In the literature, access for MIMVS has been described through lower mini sternotomy, right parasternal incision or right anterolateral mini-thoracotomy. Different techniques have been described for the right mini-thoracotomy (surgery under direct vision with video assistance, or totally thoracoscopic approach and robotic assisted approach) and it is important that the team can adapt to one technique [41]. Highly specialized centers use a peri areolar access for improved aesthetic results [42,43], but the most common incision site is the right mini thoracotomy (Figure 3). For the direct view technique, the thoracotomy incision is placed slightly more lateral compared to the totally thoracoscopic technique, in which the incision is placed more anterior and the camera is placed more laterally, directly in line with the mitral valve. Both 2D and 3D cameras can be used, of which the 3D camera provides a very realistic image of the intrathoracic space, allowing best estimation of the depth for optimal accuracy. All instruments used are specialized long shafted instruments for endoscopic cardiac surgery.

The robotic assisted approach is the most complex to learn and has a learning curve beyond mini thoracotomy [44]. In the robotic assisted technique, a 3D camera is used to visualize the chest and robotic arms are installed through ports and a variety of instruments can be inserted (e.g., scissors, diathermia, forceps). Lack of tactile feedback has not been experienced as troublesome in experienced robotic surgeons due to the realistic image with the 3D camera and the intuitive instruments with 360-degree freedom of motion. The disadvantages of this technique are more pain experience at the thoracic incisions (ports), higher costs and complex planning due to the availability of the robot [45].

### 5.3. Cardiopulmonary Bypass: Arterial Cannulation

**Figure 4:**
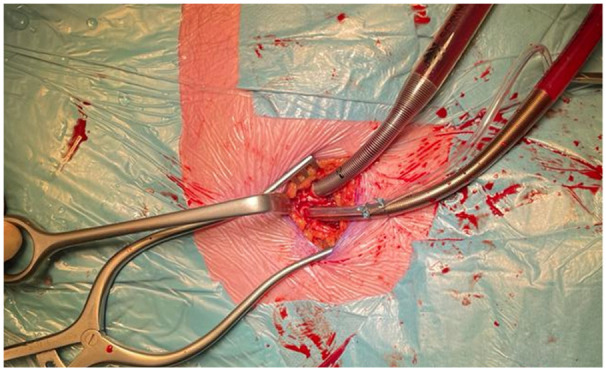
Peripheral arterial and venous cannulation in the right groin. Standard arterial cannula for direct aortic cross clamp technique. When using an endoaortic balloon, there is a sidearm in the arterial cannula to insert the balloon.

Cardiopulmonary bypass can be organized peripherally and centrally with a variety of configurations for many reasons. For peripheral cannulation, arterial access is most frequently obtained through the femoral artery (Figure 4), percutaneously (Seldinger technique) or via a groin incision, as it is a simple and reproducible technique that does not interfere with the surgical field. Whenever the femoral artery is inaccessible (e.g., in case of severe ilio-femoral calcifications, stenosis and tortuosity or thrombus in the thoraco-abdominal aorta), an 8 mm Dacron graft can be sewn on the subclavian artery for arterial cannulation [46].

For central arterial cannulation, the cannula enters the chest through the mini thoracotomy or through an extra port and is inserted directly into the aorta, comparable to traditional open cardiac surgery. The advantage of direct aortic or subclavian artery cannulation is an antegrade blood flow towards the carotid arteries which decreases the risk of stroke in patients with atherosclerotic disease or intra-aortic thrombus, in whom plaques and thrombi can be embolized during retrograde perfusion, causing stroke. However, an extra cannula in the surgical working field can interfere the surgery. A recent systematic review showed that retrograde perfusion might increase stroke risk in MIMVS compared to antegrade perfusion but has no influence on mortality and renal failure [47]. However, as mentioned by the authors, the included studies were retrospective observational studies without a pre-operative imaging protocol and therefore the association of stroke with retrograde perfusion might be biased by selection. There was only one study with a pre-operative imaging protocol, in which patients with tortuous and/or atheromatous aortoiliac-femoral arteries were selected for central cannulation. There was no difference in stroke rate of both cannulation techniques, suggesting that these patients could benefit from a modified cannulation strategy [48]. In the early experience of a center, peripheral femoral cannulation is the safest, simplest and most frequently applied technique. The pre-operative CT scan will aid in selection of patients without the complicating vascular anatomy to reduce the risk of stroke and vascular complications. In a more advanced stage, a modified cannulation strategy will expand the patient population that benefit from the minimally invasive access.

### 5.4. Cardiopulmonary Bypass: Venous Cannulation

As in arterial cannulation, peripheral venous cannulation is most frequently used in MIMVS. Access is obtained through the femoral vein (Figure 4), percutaneously (Seldinger technique) or through a groin incision, by inserting a two staged cannula positioned with the tip in the superior vena cava under TEE guidance, draining the superior and inferior caval vein. For tricuspid valve surgery or closure of an atrial septum defect, it is preferable to drain both caval veins separately, snaring both caval veins when opening the right atrium to prevent air lock in the extracorporeal circulation. When preferred an additional cannula can be inserted through the interna jugular or subclavian vein next to the femoral cannula (with the tip just below the diafragm) for separate caval venous drainage. This technique is also indicated in mitral valve surgery in patients with a vena cava filter [40] to prevent dislocation of the filter. Central venous cannulation is less frequently applied in MIMVS since it is obstructing the surgical field. It is recommended to familiarize the team with the above techniques before starting a MIMVS program. In the early experience, inserting a two staged venous cannula in the femoral vein is the simplest to learn. However, pre-operative CT planning to discover anatomical variants (e.g., Interrupted inferior vena cava or persistent left vena cava superior) and strict guidance with TEE during the insertion of the cannula is mandatory to avoid displacement injuries and to ensure adequate drainage.

### 5.5. Techniques for Aortic Occlusion and De-Airing

In the literature, there are two techniques described for aortic cross clamping in MIMVS: the endo-aortic balloon occlusion and the direct transthoracic aortic clamp (e.g., Chitwood clamp). Compared to conventional heart surgery, the surgeon is most accustomed to the direct transthoracic aortic cross-clamp and is therefore most easy to handle [49]. For the administration of cardioplegia in this technique, an aortic root vent must be placed. Removal of this vent after releasing the clamp can be challenging. One of the techniques is to remove the aortic root vent after the de-airing process and before releasing the clamp. An alternative technique is to administer retrograde cardioplegia in the coronary sinus. A catheter can be placed either by the anesthetist or the surgeon under direct view after snaring both the superior and inferior caval vein. In this technique, the patient is placed in Trendelenburg during the whole procedure and a left vent can be inserted for de-airing. Using this aorta clamp, the surgeon has to take care not to damage the left atrial appendage or the pulmonary artery. An alternative for direct aortic occlusion with a clamp is the endo-aortic balloon, which is inserted via a side branch of the arterial femoral cannula. The use of the endo balloon was recently described in detail by van Praet et al. [50,51]. In brief, the balloon occludes the aorta and cardioplegia is administered through the balloon. During the aortic occlusion, close monitoring of the pressures in the aortic root, the balloon itself and the radial artery pressure are important. Migration of the balloon is possible whenever there are changes in the pressures, such as during the start and stop of administration of cardioplegia, opening the left atrium and changes in cardiopulmonary bypass flow. In redo-surgery, the balloon can be very useful to occlude the aorta without necessity to perform adhesiolysis.

After initial concerns of stroke and aortic injuries in the early days of MIMVS by the use of the endo-aortic balloon [52], these catastrophic complications have decreased [53]. Knowledge and experience of regulating pressures in the aorta and balloon during the procedure have improved surgical outcome and studies have shown equal results compared to cross clamping [54,55]. Close communication with the perfusionist and anesthetist is necessary to prevent injuries associated with balloon migration such as stroke and aortic dissection.

When starting MIMVS, the direct transthoracic aortic clamp is the easiest to handle and for the team in training probably the safest technique. The advantage of the endo-aortic balloon is mostly in redo surgery as aortic occlusion can be obtained without adhesiolysis. However, such a technique should only be considered if the surgical team has sufficient experience in access and repair techniques to also focus on the endo-aortic balloon. Planning the procedure, it is important to choose for the technique which is the most appropriate for the surgical team experience.

### 5.6. Hypothermic Fibrillary Arrest

In selected patients, such as in redo surgery (especially in patients with patent internal mammary artery grafts after CABG), a calcified ascending aorta without safe clamping site (hostile aorta) or whenever the endo-aortic balloon is contra indicated, hypothermic fibrillary arrest can provide safe myocardial preservation. In patients with more than mild aortic valve regurgitation the vision can be hampered by retrograde blood. This can be overcome by introducing a second suction device in the left ventricle. In some patients a short stop of the heart lung machine at 25 degrees Celsius in Trendelenburg position can be very helpful to improve vision of especially the posterior annulus. De-airing can be done by leaving a vent in the left ventricle until sinus rhythm is restored and TEE confirm no residual air in the left ventricle. Experienced centers in MIMVS have reported outcomes of this technique with good results [7,8,10]. However, since this technique requires extensive surgical experience, it should not be applied during the learning curve.

### 5.7. Valve Repair and Replacement Techniques

Repair rates of MIMVS have been reported to be comparable to sternotomy approach [13,14]. All techniques for mitral valve repair are feasible through minimally invasive access, although there is a trend towards ‘respect’ rather than ‘resect’ in MIMVS. Recent studies have shown that the neochord technique showed better long-term results than the resection technique in patients with degenerative disease who underwent mitral valve repair through minimally invasive access [56,57].

## 6. Conversion to Sternotomy

**Figure 5:**
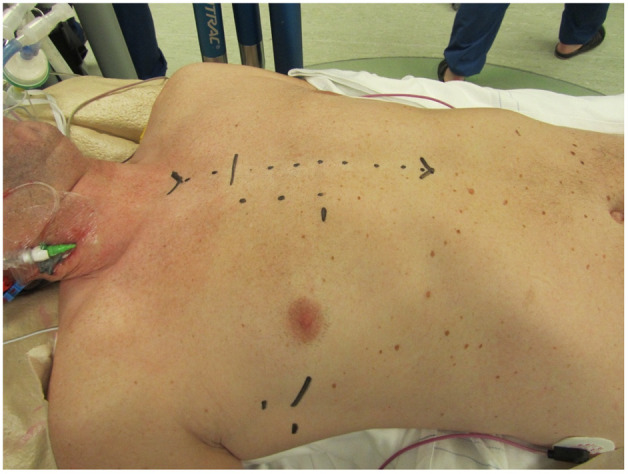
Marking of the incisions for MIMVS and the incision for conversion to sternotomy.

Conversion to sternotomy is reported at 1–2% in large series of experienced centers [4,5,6] and can be divided in an intended and unintended sternotomy. A perioperative unintended conversion is an emergency sternotomy due to a lesion of a major vascular structure or the heart (e.g., aortic dissection, atrioventricular dissociation). An intended conversion is a perioperative anticipated conversion to sternotomy due to inaccessible anatomy for MIMVS (e.g., unexpected peri-operative pulmonary adhesions). The Leipzig group have analyzed their conversions to sternotomy and the reported main reasons were major bleeding (53%), severe pulmonary adhesions (18%) and type A aortic dissection (15%) [58]. Potential bleeding site were the left atrial appendage (due to clamping) and left ventricular apex (due to the water tube for the water test). Awareness of these above-mentioned risks can prevent such complications. During the standard preparations on the operating room before minimally invasive surgery, marking of the incision for sternotomy can aid in such an emergency situation (Figure 5).

In most cases of such an emergency conversion (such as for bleeding or aortic dissection), the patient is already on cardiopulmonary bypass. In case of a major bleeding, a cardiotomy suction sump can be placed in the chest, followed by an emergency sternotomy to repair whatever is causing the bleeding. Whenever there are pulmonary adhesions, there is enough time to perform a standard sternotomy to proceed with the surgery.

## 7. Conclusions

MIMVS is increasingly adopted worldwide as the standard approach for mitral valve surgery due to less surgical trauma resulting in faster postoperative recovery and improved functional outcome. MIMVS is a complex technique with a long learning curve. The ideal case to start with is a mitral valve replacement or a straight-forward mitral valve repair without pulmonary hypertension in a non-obese patient with good left ventricular function, no mitral calcification, no chest deformity, no previous cardiac surgery and no adhesions in the right chest. The most reproducible and simplest technique is access through right mini thoracotomy (totally thoracoscopic or with video assistance and direct view), cardiopulmonary bypass through peripheral femoral arterial and venous cannulation and aortic occlusion with a direct transthoracic clamp. In a more experienced stage, more complex patients can be operated on with more complex techniques, such as the use of the endo-aortic balloon, robotic assistance and patient specific modified cannulation configurations. However, moving towards more complex techniques should never compromise operative safety and reparability of the mitral valve. The number required to complete the learning curve is currently reported at 75–125 cases and at least 2 cases per week to maintain optimal surgical outcome. Technology (such as a training simulator) can decrease this number but is not yet confirmed by studies.

## Figures and Tables

**Table 1:** Relative contraindications and concerns for MIMVS reported by the International Society for Minimally Invasive Cardiothoracic Surgery (ISMICS).

Significant Aortic, Iliac, or Femoral Disease That Prevents Safe Retrograde Arterial Perfusion
Left ventricular ejection fraction < 25%
Severe right ventricular dysfunction
Pulmonary artery pressure > 70 mmHg
Aorta > 4 cm if endo-aortic balloon being used
Significant mitral annular calcification
Patients with more than mild aortic regurgitation
Kyphoscoliosis and pectus excavatum
Morbidly obese and extremely muscular patients
Previous right thoracotomy or expected adhesions in the right chest
Advanced renal- or liver disease, significant pulmonary disease

Table derived from Ailawadi et al. 2016 [33].
